# Sodium–glucose cotransporter-2 inhibitors improve cardiovascular outcomes post-acute coronary syndrome complicated by acute heart failure

**DOI:** 10.3389/fcvm.2024.1383669

**Published:** 2024-05-20

**Authors:** Alaa Rahhal, Tahseen Hamamyh, Ammar Chapra, Khaled J. Zaza, Mostafa Najim, Mohammad Hemadneh, Hazem Faraj, Wael Kanjo, Ahmed Yasin, Haneen Toba, Wafa Mohammed, Mohammad Khair Hamad, Nawras Al-Tikrety, Mhd Baraa Habib, Ahmed Awaisu, Ahmed Mahfouz, Sumaya Alyafei, Abdul Rahman Arabi, Ashfaq Patel, Mohammed Al-Hijji

**Affiliations:** ^1^ Pharmacy Department, Hamad Medical Corporation, Doha, Qatar; ^2^ Cardiology Department, Hamad Medical Corporation, Doha, Qatar; ^3^ Anesthesiology, Intensive Care and Perioperative Medicine Department, Hamad Medical Corporation, Doha, Qatar; ^4^ Internal Medicine Department, Rochester Regional Health, New York, NY, United States; ^5^ Internal Medicine Department, Hamad Medical Corporation, Doha, Qatar; ^6^ Endocrinology Department, Hamad Medical Corporation, Doha, Qatar; ^7^ Department of Clinical Pharmacy and Practice, Qatar University, Doha, Qatar; ^8^ Interventional Cardiology Department, Hamad Medical Corporation, Doha, Qatar; ^9^ Heart Failure Department, Hamad Medical Corporation, Doha, Qatar; ^10^ Department of Medicine, Weill Cornell Medicine, Doha, Qatar

**Keywords:** acute coronary syndrome, heart failure, sodium–glucose cotransporter-2 (SGLT-2) inhibitors, HF hospitalization, cardioprotection

## Abstract

**Background:**

Acute coronary syndrome (ACS) remains a risk factor for heart failure (HF). Therefore, we aimed to assess the cardioprotective role of sodium–glucose cotransporter-2 (SGLT2) inhibitors post-ACS in patients with acute HF (AHF) and diabetes.

**Methods:**

We conducted a retrospective observational cohort study employing propensity score matching. This study involved patients with diabetes admitted with ACS complicated by AHF, defined as either new clinical HF requiring diuretics during the index admission or having an ejection fraction (EF) of <40%. The study population was divided into two groups; (1) SGLT2 inhibitor users and (2) SGLT2 inhibitor non-users. The Cox proportional hazard regression analysis was used to evaluate the outcomes.

**Results:**

A total of 465 patients (93% male; mean age, 55 ± 10 years) were included in this study. Using a 1 : 1 propensity score matching, 78 patients were included per arm with an absolute standardized difference of <0.1 for all baseline characteristics. The use of SGLT2 inhibitors resulted in lower composite outcomes of ACS, HF hospitalization, and all-cause mortality at 1 month and 12 months [1 month: 2.6% vs. 11.5%, HR = 0.20 (0.04–0.94), *p* = 0.041; 12 months: 14.1% vs. 23.1%, HR = 0.46 (0.22–0.99), *p* = 0.046].

**Conclusion:**

The findings suggest that SGLT2 inhibitors may confer cardioprotective effects in ACS-induced AHF, thereby widening the spectrum for indications of SGLT2 inhibitors.

## Introduction

Sodium–glucose cotransporter-2 (SGLT2) inhibitors are widely used oral antidiabetic medications that decrease blood glucose by enhancing the urinary excretion of glucose through the proximal convoluted tubule in the kidneys. It has been proposed that SGLT2 inhibitors may also promote the loss of sodium and water from the kidneys (1). Throughout the past decade, SGLT2 inhibitors have significantly influenced clinical practice, specifically in the management of type 2 diabetes mellitus (DM) (2). Further studies have proven the benefits of SGLT2 inhibitors beyond the management of diabetes, in the primary prevention of cardiovascular events among patients with diabetes who were at high risk of such events (3–5). Follow-up landmark randomized controlled trials have demonstrated consistent results in reducing cardiovascular mortality and heart failure (HF) hospitalization regardless of the DM status across the spectrum of left ventricular (LV) dysfunction (6–9). Consequently, SGLT2 inhibitors have recently been recognized as a cornerstone therapy in guideline-directed HF failure therapy, as recommended by clinical practice guidelines (10, 11).

Acute coronary syndrome (ACS) is the leading cause of cardiovascular death worldwide (12). Despite successful revascularization and the use of secondary prevention medications, patients with ACS remain at risk of acute and chronic HF, especially in the first month following the event (13). Therefore, patients with ACS have unmet needs to further decrease their risks of developing major adverse cardiovascular events and HF.

Landmark randomized controlled trials of SGLT2 inhibitors in HF showed favorable results in patients with established chronic HF in terms of cardiovascular mortality and HF hospitalization (6–9). However, these trials excluded subjects who had recent ACS a few months prior to enrollment. Therefore, the impact of the use of SGLT2 inhibitors among patients with ACS-induced acute HF (AHF) remains uncertain. In this study, we aimed to assess the effectiveness of early initiation of SGLT2 inhibitors post-ACS-induced AHF on short- and long-term major cardiovascular outcomes.

## Methods

### Study design, setting, and population

This retrospective cohort study was conducted at the Heart Hospital in Doha, Qatar, which is the main tertiary cardiology center within Hamad Medical Corporation (HMC), the country's principal public healthcare provider. The study was approved by the Medical Research Centre and Institutional Review Board at HMC (approval number: MRC-01-22-529). In the study, we included all patients with type 2 DM who were admitted to the Heart Hospital over 4 years between 1 June 2017 and 1 June 2021, with ACS complicated by new AHF defined as newly diagnosed HF with EF < 40% or clinical HF requiring diuretic therapy during the index admission regardless of the EF. Using a whole-population sampling approach, all patients with ischemia-induced new HF who met the eligibility criteria were included. The study population was then divided into two groups: (1) SGLT2 inhibitor users and (2) SGLT2 inhibitor non-users.

### Eligibility criteria

Patients were included in the study if they met the following criteria: (1) adult patients (≥18 years); diagnosed with type 2 DM; (2) admitted with ACS, including ST-elevation myocardial infarction (STEMI), non-ST-elevation myocardial infarction (NSTEMI), or unstable angina (UA) complicated by AHF; and (3) SGLT2 inhibitor naïve prior to the index admission. Patients were excluded if they had one of the following: (1) acute kidney injury (AKI) defined as a rise in serum creatinine by at least two times the baseline value, according to the KDIGO criteria (14), (2) chronic kidney disease (CKD) with creatinine clearance < 25 ml/min/1.73 m^2^, (3) chronic HF for at least 6 months with EF < 40% prior to the current index admission of ACS, or (4) dispensed SGLT2 inhibitor for less than their follow-up period except for discontinuation due to an adverse drug reaction (ADR).

### Outcome measures

The primary outcomes included the composite of ACS, HF hospitalization, or all-cause mortality, while the secondary outcomes included ACS, HF hospitalization, all-cause mortality, stroke, and atrial fibrillation (AF). All outcomes were evaluated within 1 month and 12 months post-discharge. Patients were followed for 12 months post-discharge after the index admission, until they developed the primary and/or secondary endpoints within 12 months of discharge, or until censoring if they were lost to follow-up during the 12-month follow-up period. Loss to follow-up was defined as failure to attend confirmed outpatient clinic appointments and/or failure to refill active prescriptions on due dates.

### Data collection procedures

The baseline characteristics, medical history, concurrent medications, and the outcomes of interest were collected from the HMC's electronic medical records system (i.e., Cerner). This was achieved by reviewing clinical care documentation during the index admission and subsequent admissions, outpatient clinic visits, and emergency visits to any HMC hospitals. Relevant diagnostic investigations performed during the follow-up period were also reviewed. Data collection was conducted from 1 June 2022 to 31 December 2022, and the relevant data were manually extracted using a pretested data collection form.

### Statistical analyses

Descriptive statistics were reported in the form of frequencies and percentages for categorical variables, mean ± standard deviation (SD) for normally distributed continuous variables, and median with interquartile range (IQR) for skewed continuous variables. Pearson's chi-squared test was used to compare categorical variables between the two study groups (i.e., SGLT2 users vs. non-users), while Student's *t*-test and Mann–Whitney *U*-test were, respectively, applied to compare normally distributed continuous variables and skewed continuous variables between the groups. The primary analysis was designed to assess through the Cox proportional hazard regression model if early initiation of SGLT2 inhibitors in ACS complicated by AHF was associated with favorable clinical outcomes. Hazard ratios (HRs) with 95% confidence intervals (CIs) were computed and presented.

A propensity score-matched model (1:1) was used to adjust for differences in baseline characteristics between SGLT2 inhibitor users and non-users. The matching was done with a caliper of 0.1. A multivariate logistic regression model was used to obtain propensity scores, with the following variables included: age, gender, Asian ancestry, smoking history, type of ACS diagnosis upon admission, percutaneous coronary intervention (PCI) performance during the index admission, medical history, ejection fraction (EF), new mitral valve regurgitation during the index admission, HbA1c value, and concurrent medications upon discharge, including dual antiplatelet therapy, statin, beta-blocker, angiotensin-converting enzyme (ACE) inhibitor/angiotensin receptor blocker (ARB), sacubitril/valsartan, aldosterone receptor antagonist, and ivabradine. The absolute mean differences were calculated for all the variables after matching with a difference of <0.1 considered as minimal match imbalance. All *p*-values were two-sided with a *p*-value of <0.05 indicating statistical significance. Data analyses were performed using the Statistical Package for Social Sciences program version 28.0 (IBM SPSS Statistics for Windows; IBM Corp., Armonk, NY, USA).

## Results

### Subject selection

**Figure 1:**
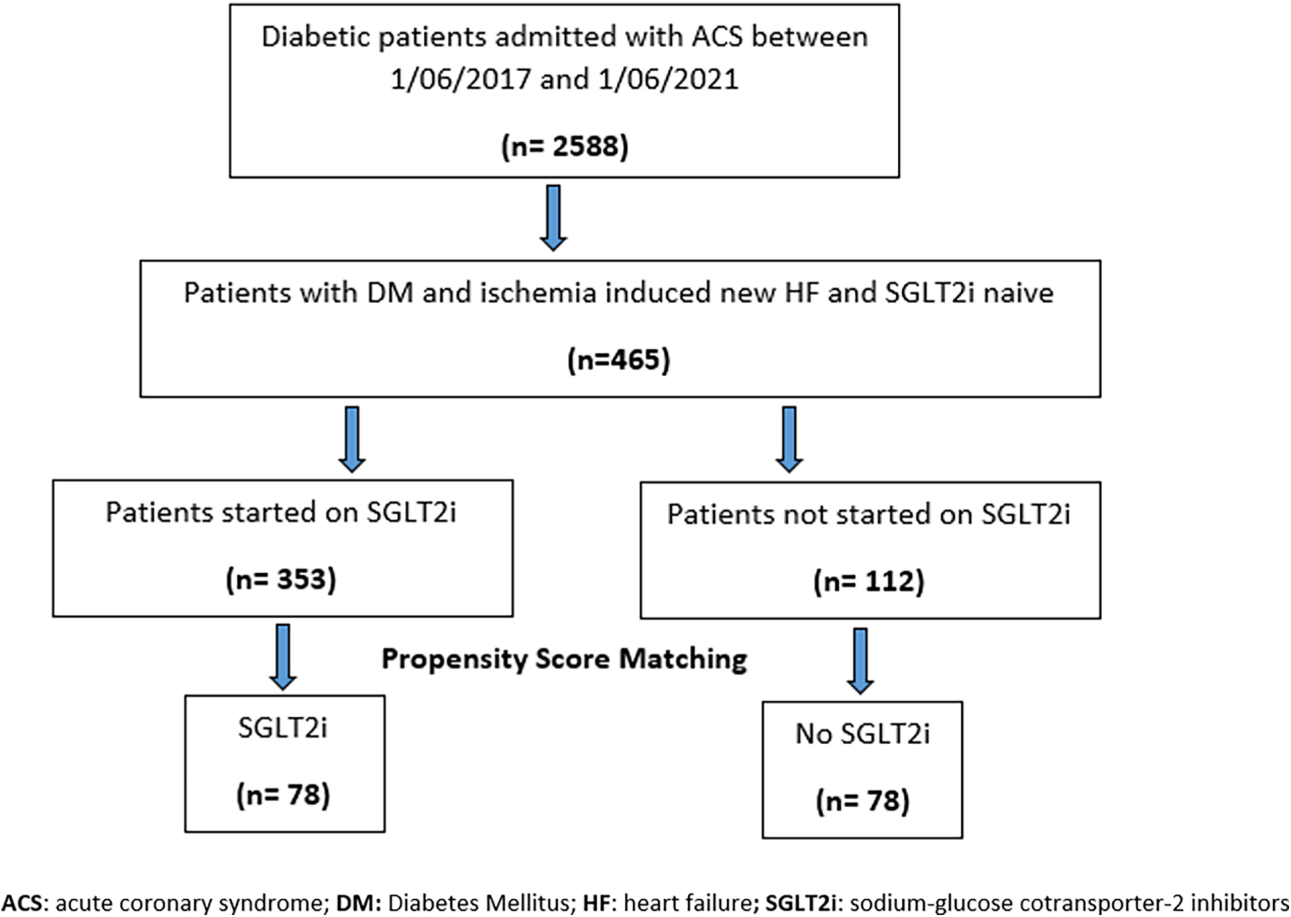
Flow chart of subjects’ enrollment.

A total of 2,588 subjects with type 2 DM who were admitted with ACS to the Heart Hospital between 1 June 2017 and 1 June 2021 were initially identified (Figure 1). A total of 465 patients with ACS-induced new-onset HF during the index admission were identified as eligible subjects, after excluding subjects with AKI, CKD with CrCl < 25 ml/min/1.73 m^2^, and known HF with reduced EF and those who dispensed SGLT2 inhibitors for less than the follow-up period post-discharge. The 465 eligible subjects were divided into two groups: SGLT2 inhibitor users (*n* = 353) vs. SGLT2 inhibitor non-users (*n* = 112). Following a 1 : 1 propensity score matching, 78 subjects were included per arm.

### Baseline characteristics

The comparison between the study groups before and after propensity score matching is presented in Table 1. In the unmatched groups, there was a significantly higher proportion of males in the SGLT2 inhibitor user arm [335 (94.9%) vs. 96 (85.7%), *p*-value = 0.001], whereas the incidence of STEMI was higher in the control arm (i.e., SGLT2 inhibitor non-user arm) [92 (82.1%) vs. 212 (60.1%), *p*-value < 0.001]. Concurrent medical conditions, including hypertension, coronary artery disease, and AF, were similar between the two groups, except for CKD, which was higher in the SGLT2 inhibitor non-user group [11 (9.8%) vs. 12 (3.4%), *p*-value = 0.006]. Similarly, EF < 30% was more frequent among the SGLT2 inhibitor non-user group [28 (25%) vs. 45 (12.7%), *p*-value = 0.002]. SGLT2 inhibitor users had significantly higher baseline HbA1c levels [HbA1c > 9%: 227 (65%) vs. 33 (30%), *p*-value < 0.001]. All concurrent medications for ACS and HF were balanced between the two groups, except for ACE inhibitors/ARBs and aldosterone receptor antagonists, which were significantly more prescribed in the SGLT2 inhibitor user arm [302 (85.6%) vs. 69 (61.6%), *p*-value < 0.001, 76 (21.5%) vs. 9 (8%), *p*-value = 0.001, respectively]. Consequently, a propensity score-matched approach was applied to adjust for these differences in baseline characteristics between the study groups.

**Table 1:** Baseline characteristics of ACS patients post-ACS-induced AHF (*n* = 465).

Characteristic	All population (*n* = 465)	Before PS matching (*n* = 465)			After PS matching (*n* = 156)			
		SGLT2i users (*n* = 353)	SGLT2i non-users (*n* = 112)	*p*-value	SGLT2i users (*n* = 78)	SGLT2i non-users (*n* = 78)	*p*-value	ASD
Age (years)	55 ± 10	55 ± 10	56 ± 10	0.229	55 ± 10	55 ± 10	0.113	0.09
Male gender	431 (92.7)	335 (94.9)	96 (85.7)	0.001	70 (89.7)	69 (88.5)	0.797	0.04
Asian ancestry	336 (72.3)	251 (71.1)	85 (75.9)	0.324	51 (65.4)	59 (75.6)	0.160	0.09
Smoking	191 (41.1)	151 (42.8)	40 (35.7)	0.186	23 (29.5)	32 (41)	0.131	0.08
Admission diagnosis								
STEMI	304 (65.4)	212 (60.1)	92 (82.1)	<0.001	54 (69.2)	64 (82.1)	0.062	0.09
NSTEMI	151 (32.5)	131 (37.1)	20 (17.9)	<0.001	23 (29.5)	14 (17.9)	0.090	0.08
PCI	358 (77)	279 (79)	79 (70.5)	0.063	56 (71.8)	59 (75.6)	0.585	0.08
Medical history								
Type II diabetes	465 (100)	353 (100)	112 (100)	NA	78 (100)	78 (100)	NA	NA
Hypertension	232 (49.9)	175 (49.6)	57 (50.9)	0.808	49 (62.8)	43 (55.1)	0.329	0.08
CKD	23 (4.9)	12 (3.4)	11 (9.8)	0.006	3 (3.8)	4 (5.1)	1*	0.06
CAD	102 (21.9)	77 (21.8)	25 (22.3)	0.910	25 (32)	15 (19.2)	0.067	0.09
Atrial fibrillation	6 (1.3)	4 (1.1)	2 (1.8)	0.594	1 (1.3)	2 (2.6)	1*	0.07
Ejection fraction ≤ 30%	73 (15.7)	45 (12.7)	28 (25)	0.002	9 (11.5)	11 (14.1)	0.632	0.07
New mitral regurgitation	142 (30.5)	116 (32.9)	26 (23.2)	0.053	17 (21.8)	20 (25.6)	0.572	0.09
HbA1c ≥ 9%	260 (56.6)	227 (65)	33 (30)	<0.001	23 (29.5)	29 (37.2)	0.308	0.08
Concurrent medications								
Dual antiplatelet	456 (98.1)	344 (97.5)	112 (100)	0.088	78 (100)	78 (100)	NA	NA
Statin	463 (99.6)	351 (99.4)	112 (100)	0.425	78 (100)	78 (100)	NA	NA
Beta-blocker	451 (97)	340 (96.3)	111 (99.1)	0.132	78 (100)	77 (98.7)	0.316	0.06
ACEi/ARB	371 (79.8)	302 (85.6)	69 (61.6)	<0.001	60 (76.9)	58 (74.4)	0.709	0.05
Sacubitril/valsartan	9 (1.9)	9 (2.5)	0 (0)	0.122*	2 (2.6)	0 (0)	0.497*	0.09
Aldosterone receptor antagonist	85 (18.3)	76 (21.5)	9 (8)	0.001	6 (7.7)	7 (9)	0.772	0.05
Ivabradine	19 (4.1)	11 (3.1)	8 (7.1)	0.095*	3 (3.8)	4 (5.1)	1*	0.06

ACS, acute coronary syndromes; AHF, acute heart failure; ASD, absolute standardized difference–a difference of <0.1 indicated minimal match imbalances; SGLT2i, sodium–glucose cotransporter-2 inhibitors; HF, heart failure; STEMI, ST-elevation myocardial infarction; PS, propensity score; NSTEMI, non-ST-elevation myocardial infarction; PCI, percutaneous coronary intervention; CKD, chronic kidney disease; CAD, coronary artery disease; ACEi/ARB, angiotensin-converting enzyme inhibitor/angiotensin receptor blocker.

* *p*-value obtained using Fisher's exact test.

The 1 : 1 propensity score matching yielded balanced groups across all baseline variables with an absolute standardized difference (ASD) of <0.1, indicating minimal match imbalances, as shown in Table 1.

### Clinical outcomes

Prior to the propensity score matching, the 1-month primary composite outcome of ACS/HF/all-cause mortality was significantly lower among the SGLT2 inhibitor users compared to the non-users as shown in Table 2 [6 (1.7%) vs. 13 (11.6%), HR 0.13 (0.05–0.35), *p*-value < 0.001]. Similarly, the 12-month primary composite outcome maintained a similar trend with nearly twice the likelihood of the hazard in the SGLT2 inhibitor non-users [35 (9.9%) vs. 26 (23.2%), HR 0.31 (0.19–0.52), *p*-value < 0.001]. Furthermore, among the secondary outcomes at 1 month, HF-related hospitalization and stroke were significantly lower in the SGLT2 inhibitor users compared to non-users [1 (0.3%) vs. 9 (8%), *p*-value < 0.001; 1 (0.3%) vs. 3 (2.7%), *p*-value = 0.045, respectively]. At 12 months, the HF hospitalization and stroke were similarly significantly lower in the SGLT2 inhibitor user group, in addition to lower AF incidence [1 (0.3%) vs. 3 (2.7%), *p*-value = 0.045].

**Table 2:** Outcomes of SGLT2i use patients with post-ACS-induced AHF (*n* = 465).

Outcomes	Before PS matching (*n* = 465)				After PS matching (*n* = 156)			
	SGLT2i users (*n* = 353)	SGLT2i non-users (*n* = 112)	HR (95% CI)	*p*-value	SGLT2i users (*n* = 78)	SGLT2i non-users (*n* = 78)	HR (95% CI)	*p*-value
Primary outcomes								
A composite of ACS, HF, or all-cause mortality at 30 days	6 (1.7)	13 (11.6)	0.13 (0.05–0.35)	<0.001	2 (2.6)	9 (11.5)	0.20 (0.04–0.94)	0.041
A composite of ACS, HF, or all-cause mortality at 360 days	35 (9.9)	26 (23.2)	0.31 (0.19–0.52)	<0.001	11 (14.1)	18 (23.1)	0.46 (0.22–0.99)	0.046
Secondary outcomes at 30 days								
HF hospitalization	1 (0.3)	9 (8)	<0.001*		0 (0)	6 (7.7)	0.028*	
All-cause mortality	3 (0.8)	0 (0)	1*		2 (2.6)	0 (0)	0.497*	
ACS	4 (1.1)	4 (3.6)	1*		2 (2.6)	2 (2.6)	1*	
Stroke	1 (0.3)	3 (2.7)	0.045*		1 (1.3)	2 (2.6)	1*	
Atrial fibrillation	0 (0)	0 (0)	NA		0 (0)	0 (0)	NA	
Secondary outcomes at 365 days								
HF hospitalization	11 (3.1)	19 (17)	<0.001		5 (6.4)	13 (16.7)	0.044	
All-Cause mortality	9 (2.5)	0 (0)	0.122*		3 (3.8)	0 (0)	0.245*	
ACS	19 (5.4)	9 (8)	0.304		8 (10.3)	5 (6.4)	0.384	
Stroke	2 (0.6)	5 (4.5)	0.010*		1 (1.3)	3 (3.8)	0.620*	
Atrial fibrillation	1 (0.3)	3 (2.7)	0.045*		1 (1.3)	1 (1.3)	1*	

SGLT2i, sodium–glucose cotransporter-2 inhibitors; HF, heart failure; PS, propensity score; HR, hazard ratio; CI, confidence interval; ACS, acute coronary syndrome; AHF, acute heart failure.

* *p*-value obtained using Fisher's exact test.

**Figure 2:**
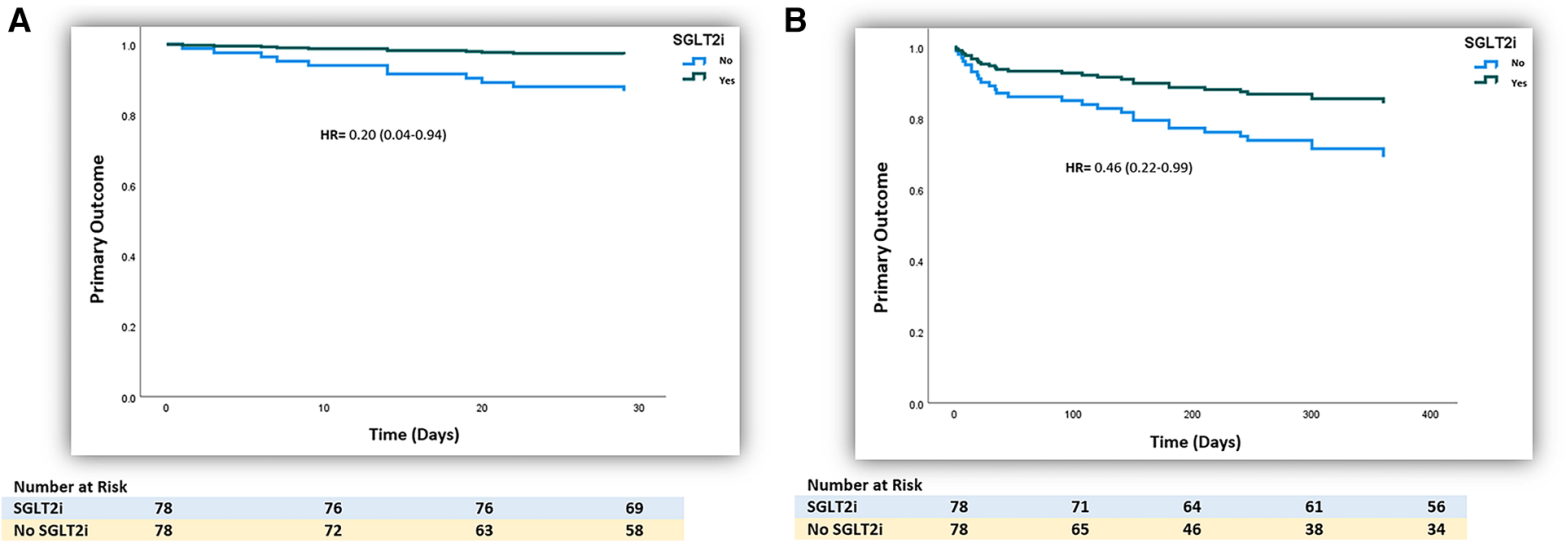
(**A**) Kaplan–Meier curve of the 1-month primary outcome of the use of SGLT2 inhibitors in patients with post-ischemia-induced new-onset heart failure after PS matching (*n* = 156). (**B**) Kaplan–Meier curve of the 12-month primary outcome of the use of SGLT2 inhibitors in patients with post-ischemia-induced new-onset heart failure after PS matching (*n* = 156).

After the propensity score matching, the primary composite outcomes remained significantly lower with the use of SGLT2 inhibitors (1 month: 2 (2.6%) vs. 9 (11.5%), HR 0.20 (0.04–0.94), *p*-value = 0.041; 12 months: 11 (14.1%) vs. 18 (23.1%), HR 0.46 (0.22–0.99), *p*-value = 0.046, respectively], as presented in Table 2 and illustrated by Kaplan–Meier curves (Figures 2A,B). The significantly lower HF hospitalization rate persisted among SGLT2 inhibitor users at 1 month and 12 months following matching, as presented in Table 2.

## Discussion

This retrospective observational study has demonstrated a significant cardiovascular benefit in patients with ACS complicated by AHF who were started on SGLT2 inhibitors upon hospital discharge. The use of SGLT2 inhibitors decreased the risk of new ischemic events, HF hospitalization, or all-cause mortality at 1 month and 12 months following discharge. These findings remained consistent among ACS patients after adjustments for baseline differences using propensity score matching.

Landmark trials evaluating the efficacy and safety of the use of SGLT2 inhibitors in HF, including DAPA-HF, EMPEROR-Reduced, EMPEROR-Preserved, and DELIVER, showed similar positive outcomes, although the studied populations were limited to patients with chronic HF, excluding those with recent ACS (6–9). The observed cardiovascular benefits in our study and the HF trials were largely driven by reduced HF-related admissions (6–9). This observation might be explained by the recently published EMPULSE trial, which investigated the clinical impact of empagliflozin use in AHF. The study showed consistent improvement in surrogate congestion parameters such as body weight and N-terminal pro-B-type natriuretic peptide (Nt-proBNP) levels for 3 months following hospital discharge (15). The EMPULSE and SOLOIST-WHF trials explored the benefits of using SGLT2 inhibitors in AHF; however, both trials excluded patients whose HF was preceded by an ACS event 3 months prior to enrollment (15, 16).

Recently, James et al*.* (17) in the DAPA-MI trial demonstrated that early initiation of dapagliflozin post-STEMI and NSTEMI with impaired left ventricular systolic function on echocardiography, or Q-wave myocardial infarction on electrocardiogram, resulted in similar cardiovascular mortality and HF hospitalization compared to placebo [2.5% vs. 2.6%, HR, 0.95; 95 CI (0.64–1.40)]. However, more than 66% of patients included in the DAPA-MI trial had EF of 30%–49%, more than 21% had an EF of ≥50%, and AHF was not accounted for, which might have contributed to the trial's findings. In contrast to the DAPA-MI trial, our inclusion criteria were selective of patients who had ACS complicated by AHF as these patients might be at higher risk for developing persistent ischemic cardiomyopathy following the acute event of ACS, necessitating the use of implantable cardioverter-defibrillator (ICD) to reduce the risk of sudden cardiac death (18). On the other hand, similar to our study's population, EMPACT-MI is an ongoing clinical trial evaluating the cardiovascular outcomes of adding empagliflozin in patients with acute myocardial infarction who developed symptoms or signs of fluid overload or a drop in EF below 45% (19).

Several studies have suggested cellular mechanisms to explain the cardiovascular benefits of SGLT2 inhibitors. In animal studies, the transient expression of SGLT2 receptors in cardiac myocytes following the use of SGLT2 inhibitors in MI is believed to decrease infarct size by preventing cellular apoptosis and decreasing oxidative stress (20, 21). Santos-Gallego et al*.* (22) demonstrated a reduced LV remodeling on echocardiography following MI in 14 non-diabetic pigs who were administered empagliflozin. The underlying mechanism was thought to be related to an increase in myocardial fuel metabolism through ketone bodies instead of glucose, which enhances energy production by myocytes and, as a result, ameliorates cardiac remodeling (22). Interestingly, the SUGAR-DM-HF trial reported an improvement in LV end-systolic and diastolic volumes when empagliflozin was used among patients with diabetes who had LV systolic dysfunction. However, no significant differences were observed in EF or LV longitudinal strain (23). In addition to LV remodeling mechanisms, SGLT2 inhibitors play a role in decreasing preload and afterload pressures. This is a result of natriuresis and the subsequent attenuation of sympathetic nervous system activity, which leads to improvement in congestion (24).

The current study has several inherent limitations given its retrospective design. First, our study population was limited to patients with DM as the study review period (1 June 2017–1 June 2021) was before SLGT2 inhibitors were considered standard HF therapy per the recently updated HF clinical practice guidelines, and hence their use was restricted to DM at the time (10, 11). Second, the use and collection of real-world data from electronic medical records under a retrospective design risk information bias. However, HMC is considered the primary healthcare provider in Qatar, and all tertiary hospitals within its network use an integrated electronic health record system; therefore, missing major outcomes was less likely. Third, safety outcomes such as ADRs due to the use of SGLT2 inhibitors were not assessed as they were not readily documented. However, patients included in our retrospective analysis were those who have dispensed SGLT2 inhibitors throughout the follow-up period, as tracked by the electronic health records dispensing manager, which could reflect proper adherence and tolerability. Fourth, no *a priori* sample size calculation was performed, yet a *post hoc* power analysis of the 1-month and 12-month primary outcomes following propensity score matching revealed a power of 70% and 75%, respectively. Finally, the positive outcomes observed in our study may be followed beyond 1 year to confirm sustainability. However, this propensity score matching retrospective cohort study demonstrated that the early initiation of SGLT2 inhibitors in ACS complicated by new-onset AHF was associated with improved cardiovascular outcomes, driven mainly by HF hospitalization benefit. We expect the results of the ongoing EMPACT-MI trial to be consistent with the current findings of this study.

## Conclusion

This study suggests that early initiation of SGLT2 inhibitors post-ACS complicated by AHF is associated with cardioprotective effects driven by the reduction in HF hospitalization. However, cautious interpretation is warranted in view of the retrospective nature of the study. The study observations may provide early evidence for broadening the spectrum of SGLT2 inhibitor indications and hence encourage clinicians to initiate SGLT2 inhibitors in patients with ACS complicated by new-onset HF.

## Data Availability

The data that support the findings of this study are available on request from the corresponding author. The data are not publicly available due to privacy or ethical restrictions.
