# Corrigendum: Sodium–glucose cotransporter-2 inhibitors improve cardiovascular outcomes post-acute coronary syndrome complicated by acute heart failure

**DOI:** 10.3389/fcvm.2025.1580343

**Published:** 2025-03-05

**Authors:** Alaa Rahhal, Tahseen Hamamyh, Ammar Chapra, Khaled J. Zaza, Mostafa Najim, Mohammad Hemadneh, Hazem Faraj, Wael Kanjo, Ahmed Yasin, Haneen Toba, Wafa Mohammed, Mohammad Khair Hamad, Nawras Al-Tikrety, Mhd Baraa Habib, Ahmed Awaisu, Ahmed Mahfouz, Sumaya Alyafei, Abdul Rahman Arabi, Ashfaq Patel, Mohammed Al-Hijji

**Affiliations:** ^1^ Pharmacy Department, Hamad Medical Corporation, Doha, Qatar; ^2^ Cardiology Department, Hamad Medical Corporation, Doha, Qatar; ^3^ Anesthesiology, Intensive Care and Perioperative Medicine Department, Hamad Medical Corporation, Doha, Qatar; ^4^ Internal Medicine Department, Rochester Regional Health, New York, NY, United States; ^5^ Internal Medicine Department, Hamad Medical Corporation, Doha, Qatar; ^6^ Endocrinology Department, Hamad Medical Corporation, Doha, Qatar; ^7^ Department of Clinical Pharmacy and Practice, Qatar University, Doha, Qatar; ^8^ Interventional Cardiology Department, Hamad Medical Corporation, Doha, Qatar; ^9^ Heart Failure Department, Hamad Medical Corporation, Doha, Qatar; ^10^ Department of Medicine, Weill Cornell Medicine, Doha, Qatar

**Keywords:** acute coronary syndrome, heart failure, sodium–glucose cotransporter-2 (SGLT-2) inhibitors, HF hospitalization, cardioprotection

A Corrigendum on Sodium–glucose cotransporter-2 inhibitors improve cardiovascular outcomes post-acute coronary syndrome complicated by acute heart failure By Rahhal A, Hamamyh T, Chapra A, Zaza KJ, Najim M, Hemadneh M, Faraj H, Kanjo W, Yasin A, Toba H, Mohammed W, Hamad MK, Altikretym N, Baraa Habib M, Awaisu A, Mahfouz A, Alyafei S, Arabi A, Patel A and Al-Hijji M. (2024) Front Cardiovasc Med. 11. doi: 10.3389/fcvm.2024.1383669

In the published article, there was an error in the Funding statement. The authors declared receiving no financial support for the research, authorship, or publication of this article. The correct Funding statement appears below.

